# Long COVID in Healthcare Workers from a Pediatric Hospital in Romania: A Cross-Sectional Study of Prevalence, Symptom Burden, and the Role of Vaccination and Reinfection

**DOI:** 10.3390/jcm14165782

**Published:** 2025-08-15

**Authors:** Maria Valentina Popa, Irina Luciana Gurzu, Claudia Mariana Handra, Cristina Mandanach, Bogdan Gurzu, Dana Elena Mîndru, Mădălina Duceac (Covrig), Mădălina Irina Ciuhodaru, Letiția Doina Duceac

**Affiliations:** 1 Doctoral School of Biomedical Sciences, “Dunărea de Jos” University of Galați, 47 Domnească Street, 800008 Galați, Romania; maria_valentina_popa@yahoo.com (M.V.P.); madalinaduceac@yahoo.ro (M.D.); 2 Department of Preventive Medicine and Interdisciplinarity, Discipline of Occupational Health, “Grigore T. Popa” University of Medicine and Pharmacy, 700115 Iasi, Romania; 3 Clinical Department V, “Carol Davila” University of Medicine and Pharmacy, 050474 Bucharest, Romania; claudia.handra@gmail.com (C.M.H.); cristina.paraschiv@drd.umfcd.ro (C.M.); 4 Department of Morfofunctional Sciences II, Faculty of Medicine, “Grigore T. Popa” University of Medicine and Pharmacy, 700115 Iasi, Romania; bgurzu@yahoo.com; 5 Department of Pediatrics, Faculty of Medicine, “Grigore T. Popa” University of Medicine and Pharmacy, 700115 Iasi, Romania; mindru.dana@umfiasi.ro; 6 Department of Mother and Child Care, “Grigore T. Popa” University of Medicine and Pharmacy, 700115 Iasi, Romania; madalina.nour@umfiasi.ro; 7 Faculty of Medicine and Pharmacy, “Dunărea de Jos” University of Galați, 47 Domnească Street, 800008 Galați, Romania; letimedr@yahoo.com

**Keywords:** long COVID, healthcare workers, occupational health, vaccination, work ability, SARS-CoV-2

## Abstract

**Background/Objectives**: Long COVID, characterized by persistent symptoms following SARS-CoV-2 infection, poses a significant occupational health concern among healthcare workers (HCWs). This study aimed to evaluate the prevalence of long COVID, symptom patterns, work-related impact, and vaccination status among healthcare personnel in a Romanian pediatric hospital. **Methods:** A cross-sectional study was conducted in 2024 among 903 hospital employees during routine occupational health assessments. Data were collected using structured questionnaires and medical records, focusing on previous SARS-CoV-2 infection, vaccination status, persistent symptoms, and functional impact. **Results:** Long COVID was identified in 28.6% of participants, with excessive fatigue (53.5%), musculoskeletal pain, respiratory difficulties, and cognitive complaints being the most common symptoms. Staff with chronic comorbidities or increased exposure risk had significantly higher rates of functional impairment. Fewer reinfections were reported among vaccinated individuals; however, vaccination was not significantly associated with the presence of long COVID symptoms. Older age and comorbidities were correlated with higher risk. **Conclusions:** The findings underline the need for long-term occupational health strategies and individualized support programs for HCWs affected by long COVID, particularly in high-risk groups.

## 1. Introduction

The COVID-19 pandemic has caused not only acute health crises but also a growing burden of long-term complications known as long COVID, now recognized as a significant global public health challenge [1,2,3].

This syndrome refers to the persistence or onset of new symptoms at least 4 weeks after acute SARS-CoV-2 infection, regardless of the initial severity of the disease, according to the CDC (Centers for Disease Control and Prevention, USA) and NICE (National Institute for Health and Care Excellence, UK) [1,4,5].

The prevalence of long COVID varies depending on the population studied and the diagnostic methodology. Current data indicate a prevalence ranging from 7.5% to 41% in non-hospitalized adults, up to 37.6% in hospitalized adults, and 2–3.5% in children [5,6]. Other estimates suggest that at least 10% of those infected have developed long COVID, with an incidence of over 25% in children and adolescents [1,7].

Long COVID is a multisystem condition with over 200 documented symptoms, most commonly fatigue, shortness of breath, cognitive dysfunction, anxiety, and musculoskeletal pain [1,2,5,7]. They often affect the ability to work effectively, particularly in medical jobs that are already physically and emotionally demanding [8,9,10]. Although the exact mechanisms—such as chronic inflammation, immune imbalance, viral persistence, and autonomic dysregulation—are still being explored [1,2,3,11,12,13,14,15,16], their impact is noticeable: impaired focus, reduced physical endurance, and mental fatigue may limit HCWs’ ability to resume their duties fully.

Risk factors for developing the syndrome include advanced age, female gender, a high number of symptoms in the first week of illness, increased body mass index, and the presence of pre-existing comorbidities [11,15].

Long COVID has led to significant physical, neuropsychological and emotional disabilities, with a major socio-economic impact globally [1]. Almost half of those affected reported impairment of social and family life, prolonged absenteeism from work, reduced work capacity and even job loss [12,17]. There is no specific treatment, and management is symptomatic and focused on recovery and personalized rehabilitation, monitoring and multidisciplinary support [11,16].

The acknowledgment of this syndrome as an emerging public health issue was necessary [18]. Long COVID has attracted the attention of the medical care sector, authorities and public health organizations, being considered a new global health crisis after the acute phase of the pandemic [5,6,17]. Resources have been allocated for re-search and management, including funding and the development of specialized clinics [17,19]. There is a rising consensus that long COVID has a substantial impact on health systems and the global economy, requiring long-term monitoring, prevention and rehabilitation strategies [5,12,17].

COVID-19 vaccination has been proven to be effective in reducing the risk of long COVID, especially when administered before infection, reducing the risk by up to 40% [20,21,22,23]. While many studies and meta-analyses report that completing a COVID-19 vaccination series before infection can lower the risk of long COVID by roughly 20–30%, the protective effect varies and is generally smaller for one-dose regimens or vaccination after infection [20,21,22,24,25,26]. Results on vaccination in people already affected by long COVID are also encouraging [27,28], although other investigations show mixed or inconclusive results—particularly regarding individuals already experiencing long COVID symptoms—and the certainty of evidence remains limited due to the predominance of observational designs and differences across viral variants [29,30].

Although COVID-19 has been widely studied worldwide, little is known about its long-term effects on healthcare workers (HCWs) in Romania, particularly those in pediatric hospitals [31]. Pediatric HCWs work in environments that present distinctive challenges: close, repeated contact with unvaccinated or partially vaccinated children, greater circulation of respiratory pathogens, and practical difficulties in consistently applying infection control measures [32,33,34]. These challenges are compounded by possible deficiencies in the implementation of prevention programs [32,33], a predominantly female workforce, and the empathic involvement inherent in pediatric care [31,35,36,37]. In Eastern Europe, a meta-analysis reported pooled prevalence rates of anxiety (30%) and depression (27%) among HCWs, signaling a significant mental health burden [38]. Published studies show that in the pediatric setting, there is high transmission of COVID-19 in intensive care units [39], widespread sleep disorders and anxiety among staff [40], and increased rates of posttraumatic stress symptoms [41]. Despite these recognized risks, robust epidemiological data on long COVID in this group covering its prevalence, symptom profile, vaccination status, and associated risk factors remain limited. Such data are essential to shape occupational health policies, support return-to-work programs, and develop tailored rehabilitation strategies [8,9,10].

To address this gap, the present study investigates the prevalence and characteristics of long COVID syndrome among HCWs in a Romanian pediatric hospital. Given the occupational vulnerabilities and limited national data, our aim was to assess the impact of persistent post-COVID symptoms on work ability and to explore the role of associated factors, including comorbidities, vaccination status, and job type.

The objectives of the study were to: (1) estimate the prevalence of long COVID among staff of the ‘Sf. Maria’ Children’s Emergency Hospital in Iași; (2) characterize the spectrum and duration of persistent symptoms; (3) assess the impact of these symptoms on work capacity and (4) identify associated factors such as acute disease history, comorbidities and vaccination status. The hypotheses were as follows: (i) long COVID significantly affects the work ability of HCWs; (ii) the risk of long COVID and the severity of manifestations are influenced by individual and professional factors, including vaccination.

## 2. Materials and Methods

We carried out a cross-sectional observational study at the ‘Sf. Maria’ Children’s Emergency Hospital in Iași, which serves as the main pediatric hospital for the North-East region of Romania. The purpose of the study, which was carried out in 2024, was to evaluate how hospital employees’ work abilities were affected by prolonged COVID syndrome.

Regular occupational health check-ups were conducted in 2024, during which 903 hospital employees were enrolled in the study. Eligible participants were staff who had worked in the hospital during the COVID-19 pandemic, attended their scheduled occupational health examination in 2024, and completed the study questionnaire in full. The cohort included doctors, nurses, stretcher bearers, nursing assistants, medical registrars, as well as auxiliary and administrative personnel.

Each participant underwent a clinical evaluation, and their vaccination status and personal history of SARS-CoV-2 infection or reinfection were confirmed using the hospital’s electronic medical records.

We collected data using a structured questionnaire put together by the hospital’s occupational health team. It was based on well-known post-COVID symptom checklists from earlier studies [42,43] and adapted to the local clinical and occupational context following expert review.

Before being implemented, the questionnaire was pre-tested in a small group of hospital staff to ensure clarity, comprehensiveness, and ease of completion.

The final questionnaire was self-administered in paper format during the occupational health visit and included both closed- and open-ended questions.

It comprised four sections: (1) personal and professional profile (age, sex, job title, years of service); (2) COVID-19 history (infection dates, number of episodes, vaccination status, hospitalization); (3) persistent symptoms (list of 36 common post-COVID manifestations); and (4) impact on health and work (perceived changes in functional capacity, absenteeism, and work limitations).

Participants categorized each symptom as mild, moderate, or severe. These scores were self-reported entirely and based on how they felt in the weeks leading up to the exam. We chose this simple three-level scale rather than combining scores because it provided a clearer picture of the variations between individuals.

Each symptom’s severity was examined on its own, as shown in Table 1. This way of working follows approaches used in other published studies [42,44], and it has been linked to how patients themselves report their daily functioning and quality of life.

All personal data were anonymized prior to analysis, and only aggregated results are presented. In collecting data, we followed institutional protocols for information security, which comply with the European Union’s General Data Protection Regulation (GDPR). Participation was entirely voluntary, and each participant gave their written informed consent. Inclusion criteria were: (1) active employment at the hospital during the study period, (2) complete occupational health records, and (3) availability of documented SARS-CoV-2 infection history. Exclusion criteria were incomplete datasets or refusal to participate.

The Ethics Committee of the “Sf. Maria” Emergency Hospital for Children in Iași, Romania granted ethical approval for the investigation (approval no. 35983, 13 December 2022). The investigation was conducted in line with the Declaration of Helsinki, with all human participant procedures meeting recognized ethical requirements, and informed consent secured in writing from all participants.

We selected only full-time healthcare workers employed at a single pediatric hospital, which helped maintain sample consistency and limited variation in occupational exposure and healthcare access.

The statistical analysis was conducted using IBM SPSS Statistics, version 25.0, at a 95% significance level. Primary processing, i.e., data systematization through centralization and grouping, led to the primary indicators, which are presented as absolute values. Based on the primary data, derived indicators were obtained through various statistical comparison, abstraction and generalization procedures. Derived indicators serve to highlight the qualitative aspects of a whole, focusing on the relationship between different parts of a group of subjects or different characteristics, interdependencies between variables. The following derived indicators, described by the ANOVA test, were used: indicators of the series of values (minimum, maximum, arithmetic mean, median), indicators of dispersion (standard deviation, standard error, coefficient of variation). We used the chi-square test (qualitative non-parametric test, compares frequency distributions), the Kruskal–Wallis correlation (qualitative non-parametric test, compares ordinal variables from three or more groups), the F (ANOVA) test used when comparing the mean values recorded in two or more groups with normal distributions, Pearson’s correlation coefficient (r) (represents the correlation between two sets of parametric values, the direct/indirect correlation being given by the sign of the coefficient), Receiver Operating Characteristic (ROC) curve (plotting the specificity/sensitivity balance as a prognostic factor). According to STROBE [45], possible biases include selection (those with severe symptoms may respond more often), memory (subjective reporting), confusion (comorbidities, vaccination) and loss to follow-up. We minimized these risks through standardized questionnaires, reminders for participation, statistical adjustments and verification of missing data, which were few.

The intercorrelation matrix, a reliability analysis scale, provides a picture of the degree of association between items. The values are useful for demonstrating that there are no problems with the construction of the respective items and that there is no high degree of similarity. The Cronbach alpha value = 0.938 is an acceptable value in relation to the threshold required (0.700) for validating the application of the questionnaire.

## 3. Results

**Figure 1:**
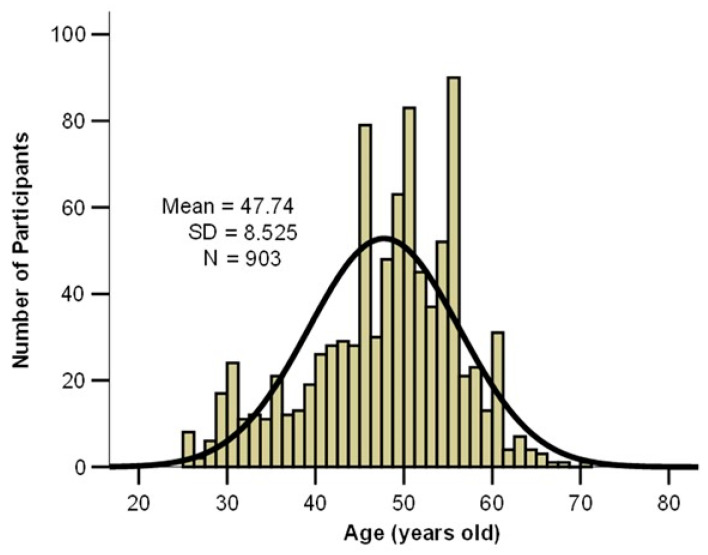
Age distribution histogram of the participants.

In the study group, 88.8% were female, with a female-to-male ratio of 7.9:1. The age of participants ranged from 25 to 71 years, with a mean age of 47.74 ± 8.525 years, close to the median of 49 years. The age distribution is approximately symmetrical, as indicated by the skewness value being greater than −2, which supports the applicability of parametric statistical tests (Figure 1).

**Figure 2:**
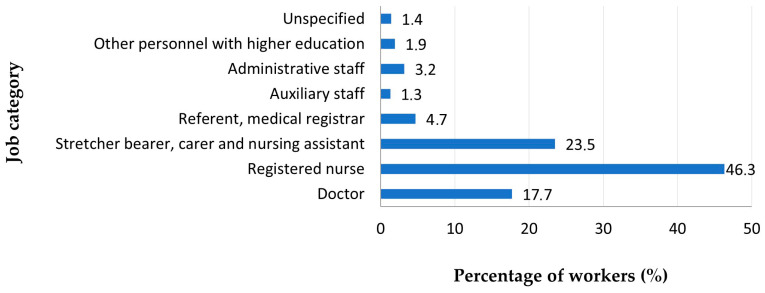
Workforce distribution by job category.

The most employees in 2024 were registered nurses (46.3%), stretcher bearers, carers and nursing assistants (23.5%) and doctors (17.7%) (Figure 2).

**Figure 3:**
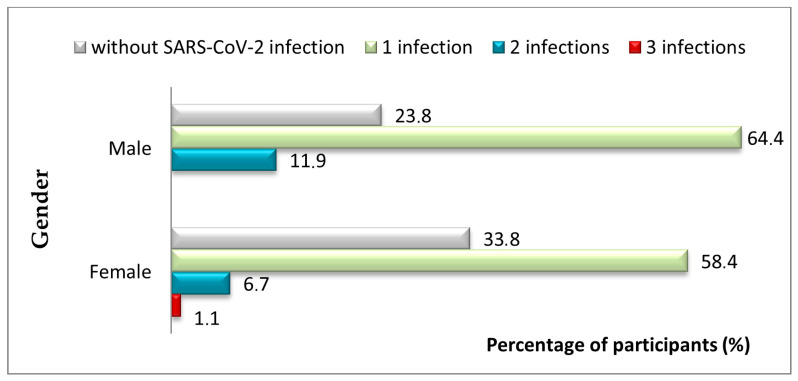
Study group structure by gender according to positive diagnosis of SARS-CoV-2 infection.

Among all workers in the study group, 67.3% of the subjects had one infection with the SARS-CoV-2 virus (59%), two infections (7.3%), or even three infections (1.0%). Regarding gender distribution (Figure 3), 23.8% of male participants were not diagnosed with SARS-CoV-2, 64.4% had one infection, and 11.9% had multiple SARS-CoV-2 infections. Among female participants, 33.8% had no infection, 58.4% had one infection, and 7.8% had two or three infections. The difference in infection frequency between genders was statistically significant (*p* = 0.037).

**Figure 4:**
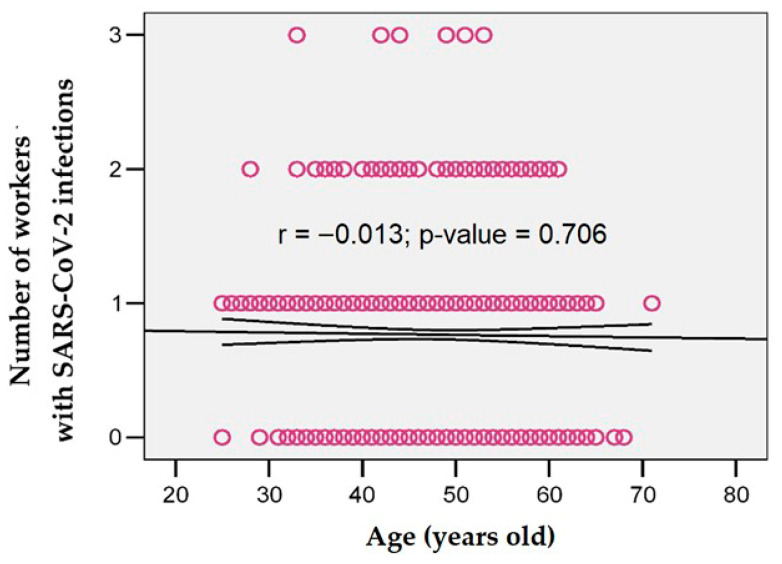
Correlation between the number of SARS-CoV-2 infections and age.

The number of SARS-CoV-2 infections did not correlate significantly with age (r = −0.013; *p* = 0.706) (Figure 4).

**Figure 5:**
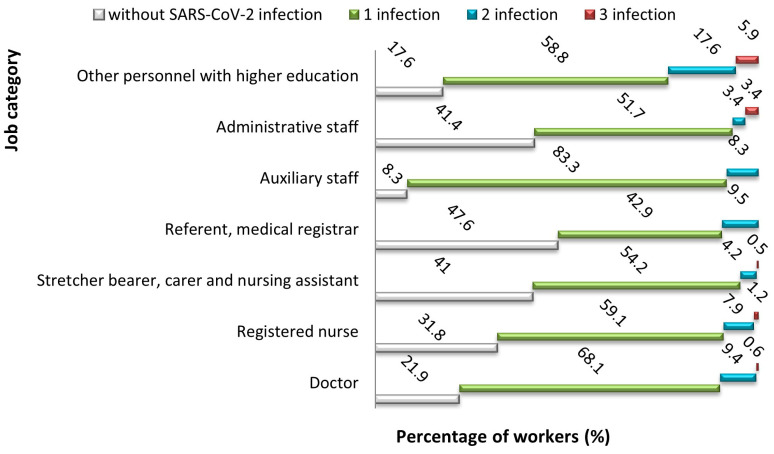
Correlation between the number of SARS-CoV-2 infections and job category.

Most subjects who were not infected with SARS-CoV-2 were medical referrers and registrars (47.6%). The percentage of cases with SARS-CoV-2 infection among auxiliary staff was 83.3%, followed by doctors (68.1%) and nurses (59.1%), but the frequency exceeded 42.9% in other employment categories as well. The number of cases with two infections was higher among staff with higher academic degrees (17.6%), medical referrers and registrars (9.5%), but also among doctors (9.4%) and auxiliary staff (8.3%). The percentage of cases with 3 infections was 1% of the total study population, but there was a percentage of 5.9% among staff with higher education and 3.4% among administrative staff (*p* = 0.009) (Figure 5).

In the study group, 15% reported that they are smokers.

Among smokers, 52.6% had one SARS-CoV-2 infection, 5.9% had two or three SARS-CoV-2 infections, and 41.5% were not diagnosed with SARS-CoV-2. Among non-smokers, the proportion of those without infection was 31.1%, 60.2% had one infection, and 7.8% had two or three infections (*p* = 0.119).

Among the 258 subjects who presented symptoms of long COVID (28.6%), 88.9% were female (*p* = 0.528), 53.9% were over 50 years old (*p* = 0.002) and 14.3% were smokers (*p* = 0.510).

**Figure 6:**
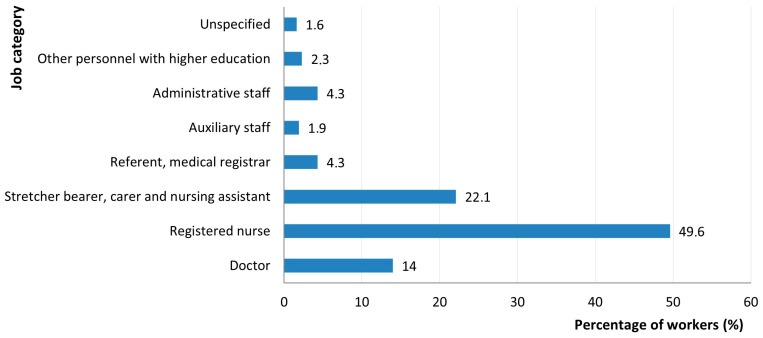
Distribution of workers with long-term effects of SARS-CoV-2 infection by job category.

The frequency of long COVID cases by work type showed the highest number of cases among nurses (49.6%; *p* = 0.009), 22.1% of care staff, 14% of doctors, and 4.3% of registrars and administrative staff (Figure 6).

The symptoms associated with long COVID syndrome varied considerably in type, intensity and duration, reflecting its multisystemic and heterogeneous clinical presentation (Table 1):

- Moderate fatigue (31%), with an average duration of approximately 11 weeks, and severe fatigue (22.5%), with an average duration of approximately 15 weeks;
- Moderate headache (12%), with an average duration of approximately 7 weeks;
- Mild loss of smell in 32.9% of patients, with an average duration of approximately 12 weeks;
- Mild loss of taste in 17.1% of patients, with an average duration of approximately 12 weeks;
- Moderate joint pain (21.3%), with an average duration of approximately 13 weeks;
- Moderate myalgia (14.7%), with an average duration of approximately 13 weeks;
- Moderate balance disorders (8.1%), with an average duration of over 11 weeks;
- Moderate dizziness (11.6%), with an average duration of approximately 10 weeks;
- Moderate dyspnea (11.2%), with an average duration of approximately 12 weeks;
- Persistent cough of moderate intensity (20.5%), with an average duration of approximately 10 weeks;
- Moderate diarrhea (3.1%), with an average duration of over 3 weeks.

Analysis of the prevalence of pre-existing chronic diseases among participants shows that, of the 266 subjects with chronic diseases (29.5% of the total sample), 87.6% were female (*p* = 0.044), 62.2% were under 50 years old (*p* = 0.001), and 16.2% were smokers (*p* = 0.208).

**Figure 7:**
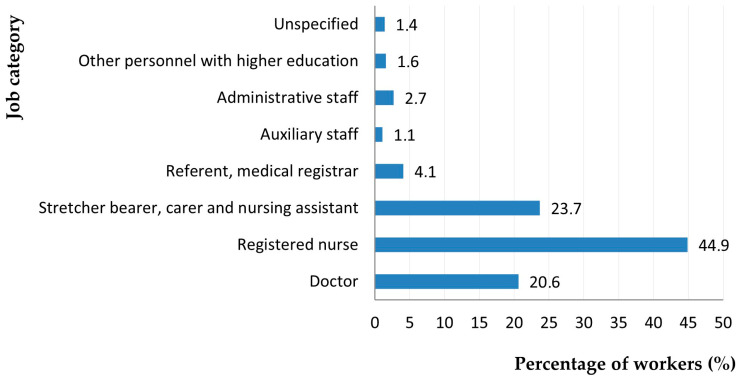
Distribution of workers with chronic diseases by occupational status.

Figure 7 shows that the highest number of cases of chronic disease was among nurses (44.9%; *p* = 0.018).

In terms of symptom progression, 393 participants (43.5%) experienced post-COVID symptoms. Of these, 3% had a complete recovery, 7% had a partial recovery, and 32.7% no longer experienced symptoms. Only 0.9% of subjects experienced worsening symptoms.

**Figure 8:**
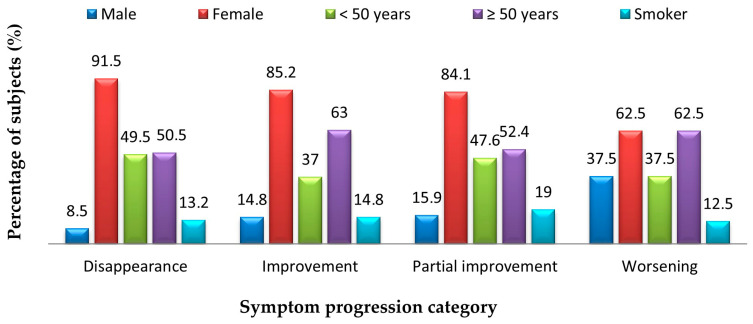
Demographic characteristics of workers based on the progression of post-COVID symptoms.

Of the eight subjects whose symptoms worsened, 62.5% were female (*p* = 0.011), 62.5% were over 50 years old (*p* = 0.022), and 12.5% were smokers (*p* = 0.841). Of the 63 subjects whose symptoms improved partially (7%), 84.1% were female (*p* = 0.011), 52.4% were over 50 years old (*p* = 0.022), and 19% were smokers (*p* = 0.841). Of the 27 subjects whose symptoms improved (3%), 85.2% were female, 63% were over 50 years old and 14.8% were smokers. Of the 295 subjects whose symptoms disappeared (32.7%), 91.5% were female, 50.5% were over 50 years old and 13.2% were smokers (Figure 8).

**Figure 9:**
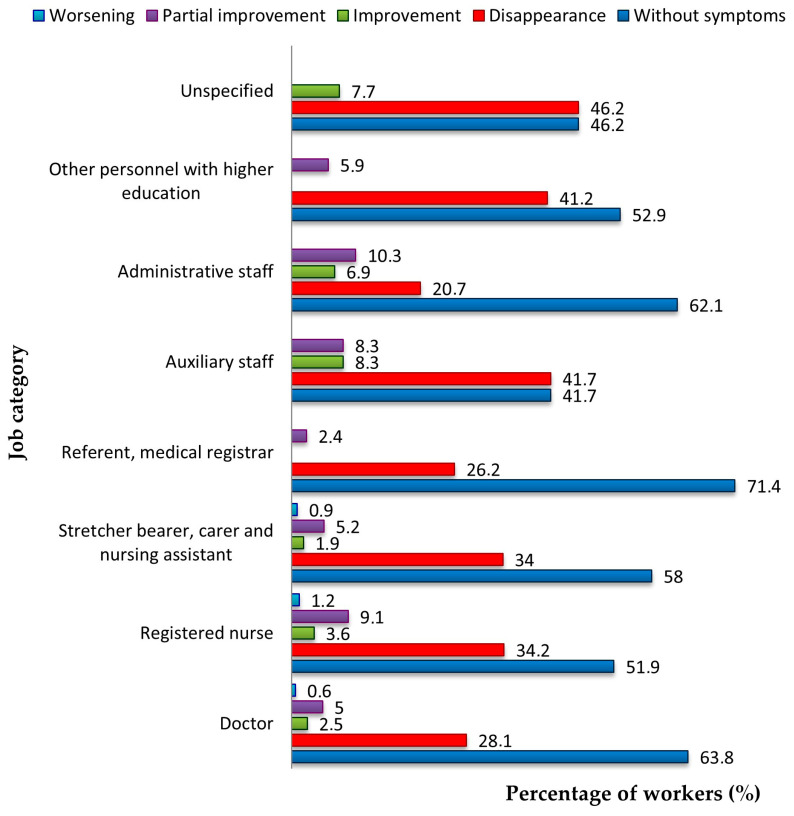
Distribution of workers by job category and symptom progression following SARS-CoV-2 infection.

We analyzed the evolution of symptoms comparatively, by function. Auxiliary staff (41.7%) and related staff with post-graduate education (41.2%) stood were those whose symptoms disappeared. In 1.2% of nurses, 0.9% of stretcher bearers, carers and nursing assistants, and 0.6% of doctors, symptoms worsened (*p* = 0.028) (Figure 9).

**Figure 10:**
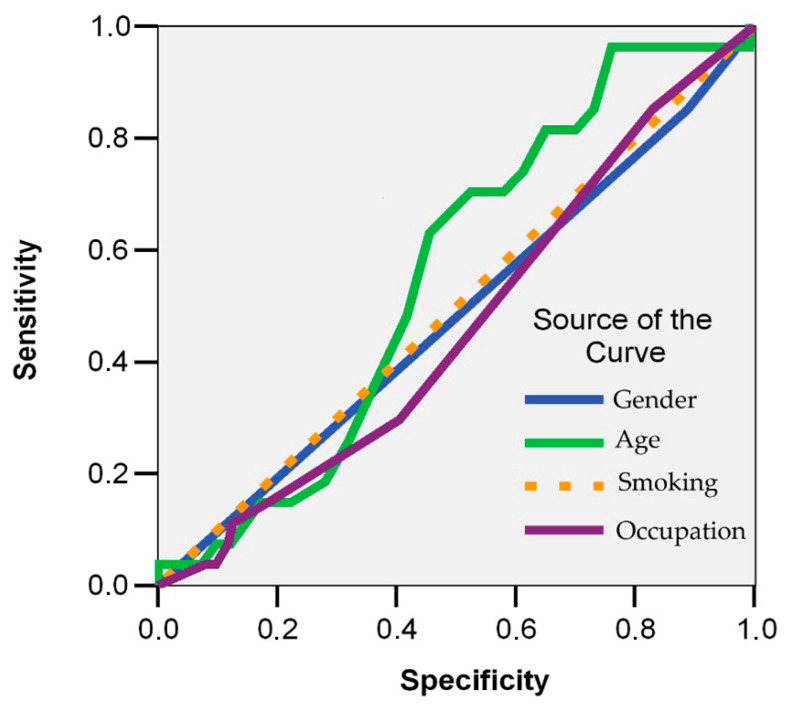
ROC curves for demographic predictors of worsening post-COVID symptoms.

Age over 45 had a moderate area under the ROC curve (AUC = 0.746; 95% CI: 0.632–0.855) among the demographic factors evaluated, with a sensitivity of 75% and a specificity of only 30%. Nevertheless, this association did not reach statistical significance (*p* = 0.099). Therefore, we exercise caution in interpreting this result and refrain from generalizing it beyond the study sample (Figure 10). We did not perform multivariable logistic regression due to the limited number of events per variable, which would have risked model overfitting. While this limits inference about independent predictors, our unadjusted analyses remain consistent with the descriptive aim of the study.

Of the total study population, 604 subjects (66.9%) were vaccinated against SARS-CoV-2: 13.1% received one dose, 34.9% received two doses and 18.9% received three doses.

Of these, 496 (82.1%) received the Pfizer/BioNTech (BNT162b2) vaccine, most commonly in two doses (51.5%). A total of 75 (12.4%) received the Johnson & Johnson (Ad26.COV2.S) vaccine in one dose, and 6 (1%) received other types of vaccine.

**Figure 11:**
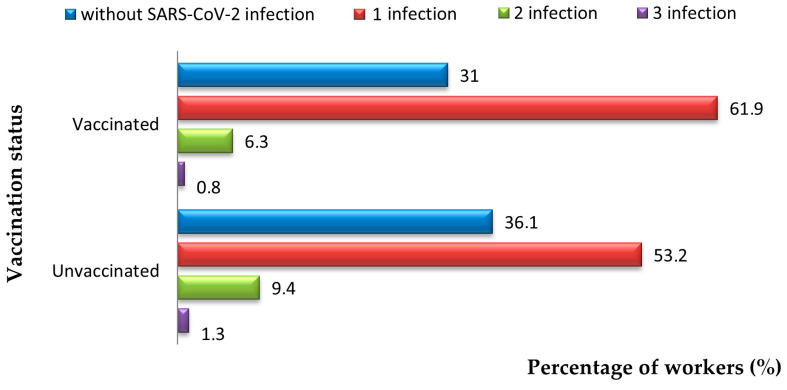
Distribution of workers vaccinated against COVID-19 correlated with positive diagnoses of SARS-CoV-2.

Of the 604 vaccinated subjects, 31% had no SARS-CoV-2 infection; 61.9% had one infection; and 7.1% had two to three infections with the SARS-CoV-2 virus. Among the 299 unvaccinated individuals, 36.1% had no SARS-CoV-2 infection, 53.2% had one infection and 10.7% had two to three infections (Figure 11).

In both the vaccinated and unvaccinated groups, the prevalence of long-term symptoms of SARS-CoV-2 infection was comparable (28.8% vs. 28.1%; *p* = 0.823).

Among the vaccinated subjects, 87.6% were female (*p* = 0.05), 53.5% were under 50 years of age (*p* = 0.375), and 15.7% were smokers (*p* = 0.203).

**Figure 12:**
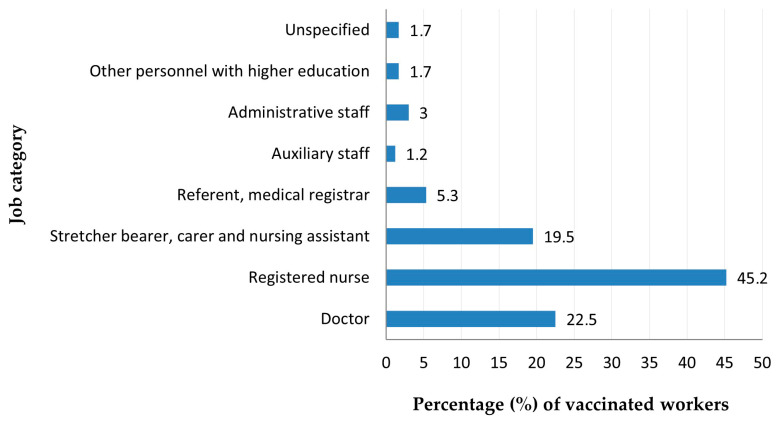
Distribution of vaccinated workers by job category.

Figure 12 shows the frequency of vaccinated subjects by job function, with the highest number of cases among nurses (45.2%; *p* = 0.001).

The most common post-COVID symptoms in vaccinated subjects were excessive fatigue (25.5%; *p* = 0.013), insomnia (11.4%; *p* = 0.025), stress (11.1%; *p* = 0.05), loss of smell (15.1%; *p* = 0.05), persistent cough (17.4%; *p* = 0.05) and eye discomfort (6.8%; *p* = 0.005) (see Table 2).

**Figure 13:**
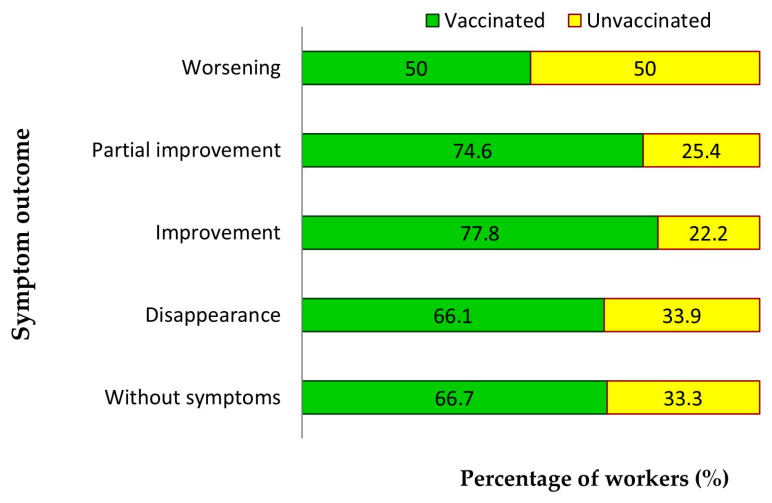
Evolution of post-COVID symptoms in relation to vaccination.

The proportion of vaccinated subjects was 66.7% among those who were asymptomatic, 66.1% among those who had recovered from their symptoms after having had the virus, 77.8% among those who had made a full recovery, and 74.6% among those who had made a partial recovery. Among subjects whose symptoms worsened after recovering from the virus, 50% were unvaccinated; however, this difference was not statistically significant (*p* = 0.340) (see Figure 13).

The prevalence of chronic diseases was similar among vaccinated and unvaccinated subjects (30.5% vs. 27.4%; *p* = 0.344), and worsening of these conditions after SARS-CoV-2 infection was reported in 5.3% of vaccinated and 6.0% of unvaccinated individuals, with no statistically significant differences (*p* = 0.380).

## 4. Discussion

More than two-thirds of the healthcare workers from the “Sfânta Maria” Children’s Emergency Hospital in Iași who took part in our study had been infected with SARS-CoV-2 at least once. Such a high rate, despite preventive measures being in place, underscores the considerable occupational exposure that pediatric healthcare staff face. It is consistent with published data indicating healthcare professionals as a high-risk group [31,35]. These findings show that healthcare systems must implement ongoing protective measures and active surveillance, especially in pediatric settings, which remain underrepresented in COVID-19 occupational health research.

Our data (Figure 3) showed that male HCWs experienced multiple SARS-CoV-2 infections more frequently than female workers, a statistically significant difference (*p* = 0.037). This result contrasts with several previous studies suggesting that female HCWs may be at higher risk of reinfection [46,47]. One possible explanation lies in the particular institutional context of our study, which was conducted in a large pediatric hospital, where patterns of exposure, job roles, and contact with infected children may differ from those in general or adult hospitals. In our pediatric hospital, male staff may have more often taken on technical or patient-facing duties that carried a higher chance of contact with COVID-19 cases. These context-specific factors could partly explain why our findings differ from those of other studies. Gaining a clearer picture of how gender influences reinfection risk in HCWs would benefit from comparisons across different hospital types and settings.

We also found no significant link between the number of infections and age (Figure 4; r = −0.013; *p* = 0.706), suggesting that being older did not necessarily increase the risk of reinfection. This aligns with trends seen in larger cohort studies. For example, a Danish follow-up study involving about 6000 HCWs reported no significant increase in infection rates among older staff members [48]. Similarly, Trujillo et al. [49] found that each additional year of age was associated with a slight reduction in the risk of symptomatic reinfection (RR per year = 0.98), while another large cohort study [50] also found no association between age and increased risk of reinfection. Instead, age appears to be more strongly associated with the burden of long COVID symptoms. As shown in the descriptive analysis presented in the Section 3, 53.9% of participants with persistent symptoms were over 50 years old (*p* = 0.002). The ROC analysis (Figure 10) showed that age over 45 had a moderate ability to distinguish individuals at risk of worsening symptoms (AUC = 0.746). We approach this result with caution, as the association failed to reach statistical significance (*p* = 0.099). Overall, our findings suggest that even if older HCWs may not be more likely to experience multiple SARS-CoV-2 infections, they might be more vulnerable to longer-lasting or more severe post-COVID symptoms.

When looking at infections by job category, auxiliary staff emerged as the most affected group (83.3%), followed by doctors (68.1%) and nurses (59.1%). This aligns with international reports showing high infection rates among nurses and other healthcare workers [35,51]. It is worth mentioning that employees in administrative or senior positions had relatively high rates of reinfection, with some suffering three distinct episodes. This pattern may be related to the dangers associated with shared offices and frequent face-to-face interactions outside of clinical tasks. Such findings point to the need for reinforcing safety protocols for non-clinical personnel as well.

Keeping pediatric healthcare staff in Eastern Europe has long been an uphill battle, shaped by chronic underfunding, heavy workloads, and too few opportunities for professional growth [52,53]. In Romania, these pressures are compounded by long-standing underinvestment in the health system, the steady emigration of medical professionals, and specific cultural barriers, all of which add to workloads, fuel burnout, and widen gaps in access to care [54,55]. Research has also identified additional sources of strain among Romanian pediatric healthcare workers, such as workplace verbal violence [56], psychological stress linked to misinformation [57], and unfavorable working conditions [58]. During the COVID-19 pandemic, pediatric emergency visits and hospital admissions in Romania dropped sharply.

However, the share of severe cases went up, hinting at delays in seeking care and a greater severity of illness at presentation [59]. This trend underlines the need for broader systemic action—such as more robust incident reporting, better conflict management training, and dedicated mental health support services for healthcare workers [60].

Our study data indicate a long COVID prevalence of 28.6%, which is comparable to rates in Brazil (27.4%) and the United Kingdom (22.5%) [51,61]. Notably, most cases of long COVID in our study were reported by women (88.9%), though this difference was not statistically significant (*p* = 0.528). However, age over 50 was significantly associated with the onset of persistent symptoms (*p* = 0.002). These findings are consistent with those of other studies indicating advanced age and female gender as risk factors [62,63].

The distribution of post-COVID symptoms by function confirmed international trends. Nurses accounted for the highest proportion of cases (49.6%; *p* = 0.009), followed by care staff (22.1%) and doctors (14%). Studies show that nurses are more prone to fatigue, complex and multisystemic, neurocognitive symptoms and depression [64,65,66]. These results correlate with the high physical and emotional demands of working in a ward dedicated to treating patients with SARS-CoV-2 infection, long shifts, and chronic stress [36,67].

Smoking was not significantly associated with the risk of infection (*p* = 0.119) or the development of long-term symptoms (*p* = 0.510), which confirms the findings of other studies that have not identified a clear relationship between this behavioral factor and disease progression [62].

The literature suggests that occupational stress, sleep deprivation, emotional overload, and a lack of organizational support can increase the vulnerability of HCWs to long-term effects of the disease and hinder recovery [68,69,70]. Furthermore, HCWs’ difficulty in accepting their status as patients contributes to stigmatization and delays access to specialized support [71]. Differences in how long COVID affected staff across occupational groups were in line with what has been reported in other studies [45,72]. Physicians tended to mention more cognitive issues, such as problems with decision-making, along with psychological distress [70,73], whereas nurses more often described fatigue, and disturbances in sleep and mood [65,74]. Auxiliary staff, meanwhile, experienced musculoskeletal pain, emotional pressure, and a lack of organizational support [75,76].

These differences support the need for a personalized approach to post-COVID monitoring and rehabilitation based on profession, age and gender. At the same time, they confirm that long COVID represents a systemic challenge for occupational health in the healthcare sector, requiring active prevention policies, psychological support, gradual reintegration and formal recognition of post-viral functional impairment [36].

These patterns can also be interpreted through the Job Demand–Resources (JD–R) model [77,78], which posits that high job demands—such as extended shifts, heavy workloads, infection risk, and emotional strain—combined with insufficient resources, including limited organizational support and inadequate recovery time, increase vulnerability to acute illness and may prolong recovery. Likewise, resilience frameworks in occupational health [79] emphasize the role of individual coping skills and institutional support systems in mitigating long-term functional impairment. Seen this way, the differences we observed in how symptoms lingered or resolved among staff with comparable infection rates might be tied to variations in the resources they could draw on and in their individual resilience [80]. Pediatric healthcare workers, in particular, face multiple challenges that can affect both resilience and occupational health, including workplace violence [81], occupational exposure [32], burnout [82,83], post-traumatic stress disorder symptoms [84], and the sustained impact of the COVID-19 pandemic [85,86].

In our study, the symptoms reported by healthcare workers with long COVID varied widely, both in how severe they were and how long they lasted. These findings are supported by data from the international literature, which describes a wide range of persistent symptoms affecting multiple organs and functions [14,87,88,89,90,91].

The most frequently reported symptom was excessive fatigue, present in 53.5% of cases (31% moderate and 22.5% severe), and the average duration increased significantly with increasing perceived severity (*p* = 0.005). These results are consistent with those reported in other studies, where fatigue is reported in 17.5–79% of HCWs [92,93,94,95]. Neurocognitive symptoms, such as memory impairment, mental exhaustion, and difficulty concentrating, were frequently reported.

The duration of memory impairment was significantly longer in moderate cases (*p* = 0.011), while the maximum duration of concentration difficulties was reached in severe cases (19.5 ± 18.94 weeks, *p* = 0.030). These data are consistent with the literature, which indicates a prevalence of cognitive disorders of 40–77% among HCWs [16,95]. Psychological symptoms such as stress, anxiety and depression were present, but there were no statistically significant differences in intensity.

However, symptom duration was prolonged, ranging from 9 to 14 weeks. Studies indicate a prevalence of anxiety and depression of 20–40% in patients with long-term effects of SARS-CoV-2 infection [16,93,94].

Neurological and sensory symptoms (headache, dizziness, paresthesia, and balance disorders) were also common. Headache was distributed across all severity grades (*p* = 0.001) and paresthesia had an increased duration in moderate cases (*p* = 0.005). The persistence of these symptoms suggests the involvement of neuroinflammatory mechanisms [90].

Musculoskeletal symptoms, such as myalgia and joint pain, were highly prevalent and distributed statistically significantly by severity (*p* < 0.05). Myalgia had a longer duration in severe forms, indicating relevant functional impairment. In some cases, SARS-CoV-2 infection was followed by an exacerbation of chronic low back pain, likely due to persistent inflammatory mechanisms, reduced physical activity, and muscle deconditioning [96]. Physical rehabilitation programs should be expanded, as these symptoms can negatively affect work capacity [97].

A significant number of patients presented with respiratory symptoms (dyspnea and persistent cough). Cough was significantly associated with severity (*p* = 0.001) and, although dyspnea had a comparable duration across all forms, it was perceived as severe more frequently (*p* = 0.001). Persistent respiratory difficulties causing medical absenteeism are one of the most common functional consequences of long-term effects of SARS-CoV-2 infection, affecting HCWs’ ability to sustain the physical activity specific to their profession and leading to repeated periods of sick leave [98].

Digestive symptoms were reported less frequently and were of low intensity. Symptoms such as nausea and abdominal pain did not show any statistically significant differences. Other long-lasting symptoms, such as hair loss, were reported after a mean period of over 20 weeks, though this was not statistically significant. This manifestation was also frequently reported in other studies on healthcare personnel [94].

The most persistent and debilitating symptoms in our cohort were fatigue, cognitive impairment, musculoskeletal pain and respiratory symptoms. These were also the most reported symptoms in international studies of healthcare personnel [16,93].

Given the high prevalence of symptoms that impact function, it is essential to implement multidisciplinary rehabilitation programs and regular clinical monitoring for affected HCWs, focusing on fatigue, cognitive symptoms, and respiratory difficulties [99,100]. Although we did not include an objective score of post-illness work capacity in our questionnaire, the negative impact is evident from the high prevalence of debilitating symptoms. Long-COVID symptoms significantly impact professional performance by affecting work capacity through fatigue, ‘brain fog’ and sleep disorders [101,102,103]. These manifestations reduce productivity, increase absenteeism and contribute to job loss or reduced working hours [104,105]. Taken together, these findings highlight the importance of recognizing long-term effects of SARS-CoV-2 infection as a risk factor for occupational dysfunction, underscoring the need for a structured, institutional approach to maintaining work capacity among HCWs [106,107].

Given their increased risk of exposure and their essential role in keeping the healthcare system running, vaccinating HCWs has been a top priority in the immunization campaign against the SARS-CoV-2 virus [108,109]. Alongside other preventive measures, such as the use of personal protective equipment, the reorganization of healthcare facilities and regular testing, the aim is to reduce transmission in healthcare settings [110]. In Romania, vaccination began in January 2021, almost ten months after the start of the pandemic, by which time many HCWs had already been infected with SARS-CoV-2.

The vaccines used in Romania included: Pfizer-BioNTech (Comirnaty), Moderna (Spikevax), AstraZeneca (Vaxzevria) and Johnson & Johnson (Janssen), with priority given to HCWs [108].

In our study, 66.9% of the 903 HCWs had received at least one dose. The most common vaccination regimens were two doses (34.9%) and three doses (18.9%), with the majority receiving the Pfizer/BioNTech vaccine (82.1%), followed by the Janssen vaccine (12.4%) and other types (1%).

The analysis of SARS-CoV-2 infections showed that 31% of vaccinated individuals were not infected, compared to 36.1% of unvaccinated individuals. Although this difference was not statistically significant, reinfections were more common in the unvaccinated group (10.7% vs. 7.1%), which suggests that vaccination provides some protection against reinfection [20,21].

Some studies have suggested that vaccination has been associated with a reduced risk of long-term symptoms of SARS-CoV-2 infection, especially when at least two doses were administered before infection [20,111]. Other studies also indicate a reduction in persistent symptoms, including fatigue and pulmonary disorders, stimulation of cellular immunity and better protection through hybrid immunity [112]. In our cohort, the prevalence of long-term symptoms was similar in vaccinated (28.8%) and unvaccinated (28.1%) individuals (*p* = 0.823), possibly because many people were infected before receiving the vaccine, limiting its protective effect against post-viral syndrome. Medical literature reports mixed results: some studies indicate partial improvement, while others show no clear effect [27,28]. For instance, an international study reported a long-COVID prevalence of 9.5% among vaccinated HCWs versus 14.6% among unvaccinated workers, who recovered faster and were less likely to be absent from work [113,114].

By job role, nurses had the highest vaccination rate (45.2%; *p* = 0.001), reflecting their prioritization in institutional campaigns. The most common post-COVID symptoms among vaccinated individuals were excessive fatigue (25.5%, *p* = 0.013), insomnia (11.4%, *p* = 0.025), stress (11.1%, *p* = 0.05), loss of smell (15.1%, *p* = 0.05), persistent cough (17.4%, *p* = 0.05), and eye discomfort (6.8%, *p* = 0.005). These results may reflect more detailed reporting by vaccinated individuals or symptoms that persisted prior to infection.

Regarding chronic diseases, the prevalence was similar between vaccinated (30.5%) and unvaccinated (27.4%) individuals (*p* = 0.344), and their worsening after COVID-19 was comparable (5.3% vs. 6.0%, *p* = 0.380), in line with international data [115,116]. In the context of occupational health, workers with chronic conditions require close monitoring, as they are a category at increased risk of post-viral complications, especially those with cardiovascular disease and diabetes mellitus [117], which are frequently associated with more severe forms of acute disease, but also with an increased risk of persistent symptoms or worsening health after infection. Vaccination in this group was an essential intervention to reduce disease severity and prevent long COVID. In workers with chronic musculoskeletal disorders [118], vaccination prevents infectious episodes that can indirectly aggravate musculoskeletal symptoms by increasing fatigue, reducing physical activity or leading to early return to work after illness.

A limitation of our study is its cross-sectional nature, which does not allow us to establish causal relationships; we can say that certain factors are associated with long COVID, but not that they directly cause it. In addition, symptom data were self-reported, which introduces possible memory bias (participants may not have accurately recalled symptom details) and reporting bias (those severely affected are more likely to respond to the questionnaire). The sample comes from a single pediatric hospital in Romania, limiting the generalizability of the results to other regions or medical specialties—pediatric hospital staff may have a different profile (younger average age, perhaps more female staff, etc.), which could influence the incidence of long COVID. Although the ROC analysis for age over 45 showed moderate discriminatory ability (AUC = 0.746), the result was not statistically significant (*p* = 0.099) and had low specificity (30%). We therefore interpret it with caution, as it suggests a possible trend but lacks sufficient validity for clinical use or generalization without further longitudinal research. It may indicate a trend but does not support clinical use or generalization without further validation in larger or longitudinal datasets. Finally, we did not have a control group (e.g., people who did not have COVID) to compare the incidence of non-specific background symptoms. However, we tried to minimize errors by using standardized definitions for symptoms and excluding incomplete responses. The limitations mentioned above call for caution in interpretation: the results indicate associations and trends, but causal relationships need to be investigated in future longitudinal studies. We did not assess occupational stress or burnout, which may be relevant factors influencing long COVID recovery and could interact with symptom persistence. In addition to these constraints, our study did not include longitudinal follow-up, lacked socioeconomic and psychological stressor assessment, and did not perform multivariable adjustment for potential confounders.

The SARS-CoV-2 vaccine has played an important role in shaping the course of the pandemic, helping to reduce severe illness, deaths, and, to some extent, the overall burden of long-term symptoms [30,31,32,33,34,35,36,37,38,39,40,41,42,43,44,45,46,47,48,49,50,51,52,53,54,55,56,57,58,59,60,61,62,63,64,65,66,67,68,69,70,71,72,73,74,75,76,77,78,79,80,81,82,83,84,85,86,87,88,89,90,91,92,93,94,95,96,97,98,99,100,101,102,103,104,105,106,107,108,109,110,111,112,113,114,115,116,117,118,119,120]. In healthcare workers, though, its effectiveness can depend on several things—when the infection occurred, which vaccine was used, how many doses were received, and whether there was any immunity beforehand. In our study, the vaccine appeared to offer some protection against reinfection but did not seem to have a major effect on the prevalence of persistent symptoms. This points to the need for more research and continued follow-up to understand the longer-term impact of vaccination in healthcare settings.

## 5. Conclusions

In our study, nearly a third (28.6%) of healthcare workers in a pediatric hospital reported lingering symptoms after COVID-19, underlining that long COVID remains a serious occupational health concern well into the post-pandemic period. The most commonly reported persistent symptoms (excessive fatigue, difficulty concentrating, breathing difficulties, and musculoskeletal pain) directly affected work ability, causing functional impairment and potentially reducing professional performance.

While our data and the literature support the important role of vaccination in reducing reinfections and severe complications, the delayed immunization of HCWs following the initial waves of the pandemic has limited the real-world ability of vaccines to prevent long-term effects. Our data confirms the need for a comprehensive approach combining prevention, sustained monitoring, and post-infection support [121,122].

From an occupational perspective, our results suggest that occupational medicine should play a central role in the early detection of persistent symptoms and the functional assessment of workers, as well as supporting the process of professional reintegration [123]. Regular occupational health consultations are an important opportunity to identify post-viral dysfunctions, refer workers to specialists and implement workplace adjustments (e.g., temporary reduction in shifts, adjustment of tasks).

The approach to long-term effects of COVID-19 among HCWs must be multidisciplinary, proactive and sustainable. It should integrate components of prevention, early diagnosis, functional support and occupational regulation. The aim is to maintain the working capacity, health and safety of HCWs.

Addressing long COVID in healthcare workers requires a mix of clinical follow-up and organizational reform.

Drawing on our findings and what is already known, we suggest making routine screening for ongoing post-COVID symptoms a standard part of occupational health check-ups. This should go hand in hand with broader access to rehabilitation and mental health support, including programs for managing fatigue and cognitive difficulties. On a broader scale, investing in workforce training and ongoing professional development may help alleviate staff shortages and improve care quality [124,125]. At the same time, increased healthcare financing is critical for attracting and retaining experienced HCWs, providing equitable compensation, and improving working conditions [54,125]. Integrating telemedicine into routine practice can extend the reach of existing personnel, particularly in underserved areas, and reduce hospital workload [125].

Hospitals and regional networks could gain from adopting more flexible staffing arrangements, sharing resources, and shifting certain tasks to help spread workloads more evenly and make better use of available staff [126]. Providing community clinics with extra training and resources might also ease pressure on hospitals by cutting down on avoidable referrals. To retain staff, targeted measures should address mental health needs, improve work–life balance, and reduce burnout [54], while also countering factors that drive emigration. Strengthening organizational support, resource allocation, and targeted interventions has been shown to mitigate negative health outcomes and improve well-being among pediatric healthcare workers [127,128,129,130,131].

## Figures and Tables

**Table 1:** Intensity and duration of symptoms in employees with long COVID.

Symptom	Symptom Severity in Total Long COVID Cases (n = 258)						*p* Chi Square Test
	Mild		Moderate		Severe		
	n	%	n	%	n	%	
Excessive fatigue Duration, weeks	24	9.3	80	31.0	58	22.5	**0.001**
	8.67 ± 5.83		10.83 ± 7.87		15.09 ± 11.57		**0.005**
Memory impairment Duration, weeks	26	10.1	21	8.1	6	2.3	0.314
	7.60 ± 5.32		12.95 ± 6.77		9.67 ± 2.66		**0.011**
Mental exhaustion Duration, weeks	19	7.4	36	14.0	13	5.0	0.651
	7.33 ± 4.50		13.03 ± 8.70		16.15 ± 10.66		**0.026**
Concentration difficulties Duration, weeks	42	16.3	51	19.8	8	3.1	0.497
	10.57 ± 8.20		11.44 ± 6.35		19.50 ± 18.94		**0.030**
Insomnia Duration, weeks	17	6.6	35	13.6	15	5.8	0.395
	9.63 ± 7.05		7.47 ± 5.73		12.93 ± 10.50		0.064
Stress Duration, weeks	12	4.7	40	15.6	21	8.1	0.598
	9.67 ± 6.97		11.41 ± 11.39		14.10 ± 9.90		0.458
Depression Duration, weeks	13	5.0	19	7.4	9	3.5	0.126
	11.83 ± 6.74		10.82 ± 6.56		9.78 ± 3.83		0.743
Anxiety Duration, weeks	18	7.0	23	8.9	11	4.3	0.821
	10.59 ± 5.86		10.62 ± 6.76		12.18 ± 4.77		0.751
Headache Duration, weeks	19	7.4	31	12.0	23	8.9	**0.001**
	8.44 ± 6.42		7.17 ± 4.70		8.82 ± 4.57		0.483
Change in smell Duration, weeks	25	9.7	33	14.0	15	5.8	0.113
	11.00 ± 7.08		12.66 ± 9.85		15.00 ± 14.59		0.497
Loss of smell Duration, weeks	85	32.9	0	0.0	0	0.0	**0.001**
	11.92 ± 9.62		-		-		-
Change in taste Duration, weeks	16	6.2	17	6.6	13	5.0	0.611
	10.20 ± 6.46		11.94 ± 5.39		14.00 ± 14.12		0.546
Loss of taste Duration, weeks	44	17.1	0	0.0	0	0.0	**0.001**
	11.75 ± 2.21		-		-		-
Joint pain Duration, weeks	28	10.9	55	21.3	40	15.5	**0.012**
	10.48 ± 5.46		13.19 ± 14.18		13.76 ± 2.49		0.581
Myalgia (muscle pain) Duration, weeks	19	7.4	38	14.7	26	10.1	**0.001**
	7.56 ± 4.16		12.84 ± 2.68		15.08 ± 3.60		**0.037**
Generalized pain Duration, weeks	14	5.4	36	14.0	12	4.7	0.902
	9.38 ± 3.86		11.03 ± 8.01		9.75 ± 5.46		0.710
Balance disorders Duration, weeks	12	4.7	21	8.1	11	4.3	**0.050**
	11.83 ± 3.75		11.45 ± 9.29		10.73 ± 3.84		0.972
Dizziness Duration, weeks	25	9.7	30	11.6	10	3.9	**0.050**
	10.33 ± 5.95		9.90 ± 7.11		6.80 ± 3.43		0.308
Paresthesia (tingling/numbness) Duration, weeks	10	3.9	13	5.0	4	1.6	0.251
	11.40 ± 6.26		17.38 ± 15.15		8.50 ± 4.12		**0.005**
Cold extremities Duration, weeks	14	5.4	13	5.0	9	3.5	0.165
	10.43 ± 4.59		8.33 ± 6.20		8.67 ± 2.83		0.511
Shortness of breath Duration, weeks	20	7.8	29	11.2	24	9.3	**0.001**
	10.84 ± 3.18		11.69 ± 9.25		12.25 ± 7.40		0.903
Persistent cough Duration, weeks	22	8.5	53	20.5	26	10.1	**0.001**
	14.67 ± 6.83		9.50 ± 5.86		9.41 ± 5.99		0.165
Palpitations Duration, weeks	16	6.2	22	8.5	13	5.0	0.484
	7.47 ± 5.87		11.90 ± 3.10		10.62 ± 5.68		0.445
Chest pain Duration, weeks	11	4.3	18	7.0	14	5.4	0.309
	7.10 ± 4.38		8.22 ± 6.10		8.64 ± 4.60		0.733
Sore throat Duration, weeks	19	7.4	15	5.8	9	3.5	0.777
	5.37 ± 4.13		4.80 ± 2.91		7.44 ± 4.28		0.248
Diarrhea Duration, weeks	5	1.9	8	3.1	5	1.9	**0.045**
	3.00 ± 3.08		3.25 ± 2.25		4.60 ± 3.29		0.618
Nausea Duration, weeks	10	3.9	9	3.5	6	2.3	0.689
	5.00 ± 2.65		4.78 ± 1.86		6.50 ± 4.18		0.493
Vomiting Duration, weeks	6	2.3	2	0.8	4	1.6	0.344
	2.17 ± 1.42		2.50 ± 2.12		5.00 ± 3.46		0.223
Loss of appetite Duration, weeks	21	8.1	31	12.0	18	7.0	0.843
	8.71 ± 10.00		10.06 ± 6.42		8.00 ± 5.93		0.628
Constipation Duration, weeks	2	0.8	5	1.9	1	0.4	0.292
	4.50 ± 4.95		6.00 ± 4.32		8.00 ± 0.00		0.821
Abdominal pain Duration, weeks	15	5.8	10	3.9	7	2.7	0.813
	5.73 ± 3.83		5.00 ± 2.75		6.29 ± 3.73		0.750
Hair loss Duration, weeks	7	2.7	24	9.3	12	4.7	0.157
	14.86 ± 8.86		20.13 ± 14.89		22.33 ± 12.59		0.509
Skin rashes Duration, weeks	17	6.6	9	3.5	1	0.4	0.460
	9.76 ± 10.49		10.25 ± 5.99		8.00 ± 0.00		0.973
Itching (pruritus) Duration, weeks	8	3.1	9	3.5	0	0.0	0.500
	8.67 ± 2.73		8.75 ± 4.53		-		0.969
Eye discomfort Duration, weeks	22	8.5	17	6.6	3	1.2	0.589
	10.55 ± 9.90		8.93 ± 3.77		8.00 ± 0.00		0.764
Fever > 38 °C Duration, weeks	25	9.7	0	0.0	0	0.0	0.901
	1.40 ± 1.41		-		-		-

**Table 2:** Distribution of cases with post-COVID symptoms according to vaccination status.

Post-COVID Symptom	Vaccinated (n = 604)		Unvaccinated (n = 299)		Chi2 Test *p*
	n	%	n	%	
Excessive fatigue	154	25.5	54	18.1	**0.013**
Memory disorders	48	7.9	19	6.4	0.390
Mental exhaustion	60	9.9	19	6.4	0.073
Difficulty concentrating	85	14.1	33	19.0	0.203
Insomnia	69	11.4	20	6.7	**0.025**
Stress	67	11.1	21	7.0	**0.050**
Depression	39	6.5	11	3.7	0.086
Anxiety	46	7.6	20	6.7	0.615
Headache	79	13.1	27	9.0	0.076
Change in smell	62	10.3	21	7.0	0.962
Loss of smell	91	15.1	33	11.0	**0.050**
Myalgia	87	14.4	31	10.4	0.091
Generalized pain	50	8.3	20	6.7	0.401
Balance disorders	36	6.0	16	6.4	0.712
Dizziness	59	9.8	23	7.7	0.307
Paresthesia	24	4.0	9	3.0	0.468
Cold extremities	33	5.5	9	3.0	0.100
Shortness of breath	69	11.4	25	8.4	0.156
Persistent cough	105	17.4	37	12.4	**0.050**
Palpitations	46	7.6	18	6.0	0.379
Sore throat	47	7.8	15	5.0	0.122
Diarrhea	23	3.8	7	2.3	0.247
Nausea	30	5.0	8	2.7	0.107
Vomiting	15	2.5	2	0.7	0.059
Loss of appetite	61	10.1	29	9.7	0.850
Constipation	7	1.2	2	0.7	0.486
Abdominal pain	31	5.1	8	2.7	0.088
Hair loss	32	5.3	16	5.4	0.974
Skin rashes	23	3.8	9	3.0	0.541
Itching (pruritus)	13	2.2	5	1.7	0.627
Eye discomfort	41	6.8	7	2.3	**0.005**
Fever > 38 °C	37	6.1	12	4.0	0.121

## Data Availability

All data from the first author and the corresponding author are available.

## Abbreviations

The following abbreviations are used in this manuscript:

%	Percentage
ANOVA	Analysis of Variance
AUC	Area Under the Curve
CI	Confidence Interval
COVID-19	Coronavirus Disease 2019
HCWs	Healthcare Workers
*p*-value	Probability Value (in statistical significance testing)
n	variable
r	Pearson’s correlation coefficient
ROC curve	Receiver Operating Characteristic curve
SD	Standard Deviation
SPSS	Statistical Package for the Social Sciences
